# The Exploration of Natural Compounds for Anti-Diabetes from Distinctive Species *Garcinia linii* with Comprehensive Review of the Garcinia Family

**DOI:** 10.3390/biom9110641

**Published:** 2019-10-23

**Authors:** Ting-Hsu Chen, May-Jywan Tsai, Yaw-Syan Fu, Ching-Feng Weng

**Affiliations:** 1 Department of Life Science and Institute of Biotechnology, National Dong Hwa University, Hualien 97401, Taiwan; 410613031@gms.ndhu.edu.tw; 2 Neural Regeneration Laboratory, Department of Neurosurgery, Neurological Institute, Taipei Veterans General Hospital, Taipei 11221, Taiwan; mjtsai2@vghtpe.gov.tw; 3 Department of Biomedical Science and Environmental Biology, Kaohsiung Medical University, Kaohsiung City 80708, Taiwan; m805004@kmu.edu.tw; 4 Institute of Respiratory Disease, Department of Basic Medical Science, Xiamen Medical College, Xiamen 361023, China

**Keywords:** *Garcinia linii*, hypoglycemia, benzopyran, triterpene, bioflavonoid, phenolic, *in silico*

## Abstract

Approximately 400 Garcinia species are distributed around the world. Previous studies have reported the extracts from bark, seed, fruits, peels, leaves, and stems of *Garcinia mangostana, G. xanthochymus*, and *G. cambogia* that were used to treat adipogenesis, inflammation, obesity, cancer, cardiovascular diseases, and diabetes. Moreover, the hypoglycemic effects and underlined actions of different species such as *G. kola*, *G. pedunculata*, and *G. prainiana* have been elucidated. However, the anti-hyperglycemia of *G. linii* remains to be verified in this aspect. In this article, the published literature was collected and reviewed based on the medicinal characteristics of the species Garcinia, particularly in diabetic care to deliberate the known constituents from Garcinia and further focus on and isolate new compounds of *G. linii* (Taiwan distinctive species) on various hypoglycemic targets including α-amylase, α-glucosidase, 5′-adenosine monophosphate-activated protein kinase (AMPK), insulin receptor kinase, peroxisome proliferator-activated receptor gamma (PPARγ), and dipeptidyl peptidase-4 (DPP-4) via the molecular docking approach with Gold program to explore the potential candidates for anti-diabetic treatments. Accordingly, benzopyrans and triterpenes are postulated to be the active components in *G. linii* for mediating blood glucose. To further validate the potency of those active components, *in vitro* enzymatic and cellular function assays with *in vivo* animal efficacy experiments need to be performed in the near future.

## 1. Introduction

### 1.1. Impact of Diabetes

Diabetes mellitus (DM) is a global health issue due to its high risk factors, e.g., obesity, physical inactivity, ageing, bad eating habits, genetic predisposition, hypertension, and hyperlipidemia [1]. It is worth noting that DM is a metabolic disease and more than 400 million people suffered from diabetes in 2014 [2]. Interestingly, the adult diabetic population of 2014 has risen from 4.7% to 8.5% worldwide in contrast to the population in 1980 [2]; the morbidity and mortality were 4.95% and 4.00%, respectively [3]. In the future, the diabetic population will increase to 642 million by around 2045 and this population will continue to grow [3].

### 1.2. Therapy Agent of Diabetes

Nowadays, many clinical medicines such as *α*-glucosidase and *α*-amylase inhibitors such as Acarbose are applied to delay the metabolism of carbohydrates and control post-meal blood glucose for diabetes patients [4]. Metformin and 5-Amino-4-Imidazolecarboxamide Riboside (AICAR) are activators that increase the glucose absorption of skeletal muscles and inhibit gluconeogenesis of the liver [5,6]. The major anti-diabetes functions of Sitagliptin increase concentrations of incretin. Incretin can further potentiate the pancreas to produce insulin and inhibit the production of glucagon to decrease blood glucose levels [7]. Rosiglitazone can reduce the resistance of insulin absorption in liver cells, skeletal muscle cells, and fatty tissues [8]. Additionally, GW-9662 is an agonist that promotes peroxisome proliferator-activated receptors gamma (PPARγ) expression, which could stimulate fat metabolism, and trigger insulin pathways to regulate blood glucose and has anti-inflammatory properties [9].

### 1.3. Distribution of Garcinia Plants and Recent Discovery of Anti-Diabetic Agents with Garcinia Plants

Approximately 400 Garcinia species are distributed around the world including Bangladesh, China, India, Indonesia, Taiwan, Thailand, tropical Asia, Southern Africa, and Western Polynesia. Usually, the Garcinia plants are shrubs or trees. Most of their edible fruits are used in agricultural societies or the fruits and seeds are used to produce oils and dyes, and to treat various diseases, e.g., abdominal pain, food allergies, arthritis, diarrhea, dysentery, and wound infections as past research has shown [10,11,12,13,14,15,16]. Most of the trunks from the Garcinia plants such as *Garcinia subelliptica* have been used as building materials to prevent destruction by typhoons in ancient Japan, which is in contrast to why *G. subelliptica* were planted as alley trees, in gardens, and as decorative plants [17,18]. Moreover, East South Asian peoples usually eat the fruit of *G. mangostana, G. xanthochymus*, and *G. cambogia* for calories or nutrition and use the extracts of *G. xanthochymus* and *G. cambogia* in curry powder to increase the sour flavor in India. Interestingly, the extract of *G. cambogia* is also used as an antiseptic for preserving food freshness [18,19].

The accumulated literature showed that metabolic syndromes gradually became public health problems such as obesity, hyperglycemia, hyperlipidemia, and hypertension and lead to cardiovascular diseases (i.e., atherosclerosis, stroke, or peripheral artery disease) or diabetes [20]. Hereafter, the medicinal plants or herbs have important characteristics that resist the threat of these diseases because the adverse side effects of natural compounds isolated from medicinal plants are reduced and often less severe than those from clinical drugs [21]. Interestingly, previous studies have reported that the extracts from the bark, seeds, fruits, peels, leaves, and stems of *G. mangostana, G. xanthochymus*, and *G. cambogia* are used to treat adipogenesis, inflammation, obesity, cancer, cardiovascular disease, and diabetes. Furthermore, these extracts could also trigger the myotubes and skeletal cells to absorb glucose and to balance blood glucose levels [20,22,23,24,25,26]. Remarkably, numerous studies have indicated that the Garcinia species, e.g., *G. cambogia* [10], *G. xanthochymus* [11], *G. kola* [12], *G. mangostana* [13,14], *G. pedunculata* [15], and *G. prainiana* [16] contain plenty of biflavonoids and phenolic compounds. These compounds have been found to inhibit the enzymatic activity of α-amylase and α-glucosidase for propagating an anti-diabetic effect [27]. Concurrently, the variability of biological actions includes anti-diabetic agents that are dependent on the constituents of plants that grow locality, differently used parts (root, leaves, flowers), or seasonal harvests that exhibit various compositions or ratios for each component, metabolite, or derivative including different delivery systems such as powers, pills, or teas, among others. Generally, batch to batch control or a standard operating system via index chemicals or fingerprints is crucial for the evaluation of the efficacy of crude extracts, the yields in isolation, and the purification of active ingredients. In this article, the published articles were collected and reviewed. We have addressed the isolated compounds for all Garcinia species by molecular structure and briefly described their targeted biomolecules for anti-diabetic function according to chemical category via PubChem such as benzophenone (one compound isolated from *G. mangostana*), biflavonoids (one compound isolated from *G. mangostana* and seven compounds isolated from *G. kola*), xanthones (forty-four compounds isolated from *G. mangostana*, thirty-two compounds isolated from *G. xanthochymus*, and nine compounds isolated from *G. hanburyi*), and procyanidin (one compound isolated from *G. mangostana*). The medicinal characteristics of the Garcinia species for the care of DM (Table 1) are listed to deliberate on the potential constituents of certain Garcinia species. Interestingly, *G. linii* is one endemic evergreen tree only distributed in outlying islands—Langyu land and Green island of Taiwan. To the best of our knowledge, active constituents including 15 xanthones, 6 biphenyls, 2 benzopyran, and 13 known compounds isolated from the root of *G. linii* have been reported with anti-tubercular activity and cytotoxicity [28,29]. However, the medicinal values of *G. linii* extract as an anti-diabetic agent remain to be explored. Moreover, this review article simultaneously offers insights into dissecting the molecular mechanism of isolated compounds such as the three new xanthones (linixanthones A–C), five new biphenyls (garcibiphenyls A–E), and two new benzopyran (garcibenzopyran and (*S*)-3-hydroxygarcibenzopyran) of the *G. linii* root from Taiwan [28,29], combined with the nine known xanthones (10-*O*-Methylmacluraxanthone; 1,5-Dihydroxyxanthone; 1,6-Dihydroxy-3,5,7-trimethoxyxanthone; 1,6-Dihydroxy-5-methoxyxanthone; 1,6-dihydroxy-5,7-dimethoxyxanthone; 1,6-Dihydroxy-7-methoxyxanthone; 1,7-Dihydroxyxanthone; 1,7-Dihydroxy-3-methoxyxanthone; 5-Hydroxy-1-methoxyxanthone) from the Garcinia family on various hypoglycemic targets including α-amylase, α-glucosidase, 5′-adenosine monophosphate-activated protein kinase (AMPK), insulin receptor kinase (IRK), PPARγ, and dipeptidyl peptidase-4 (DPP4) via the molecular docking approach with Gold program (Cambridge Crystallographic Data Centre, Cambridge, UK).

### 1.4. α-Amylase and α-Glucosidase

**Figure 1:**
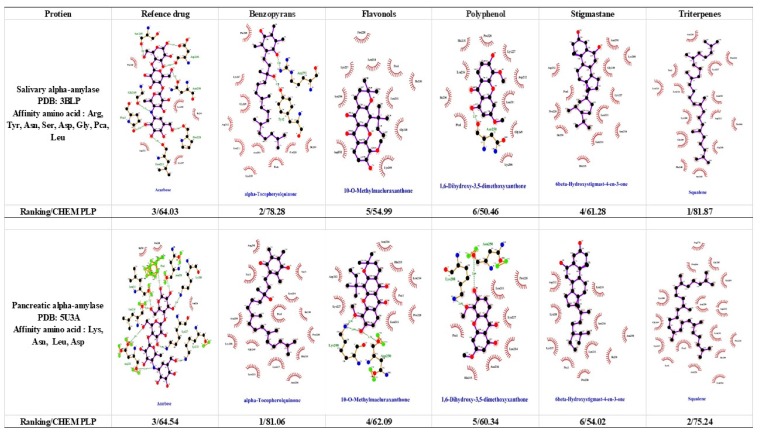

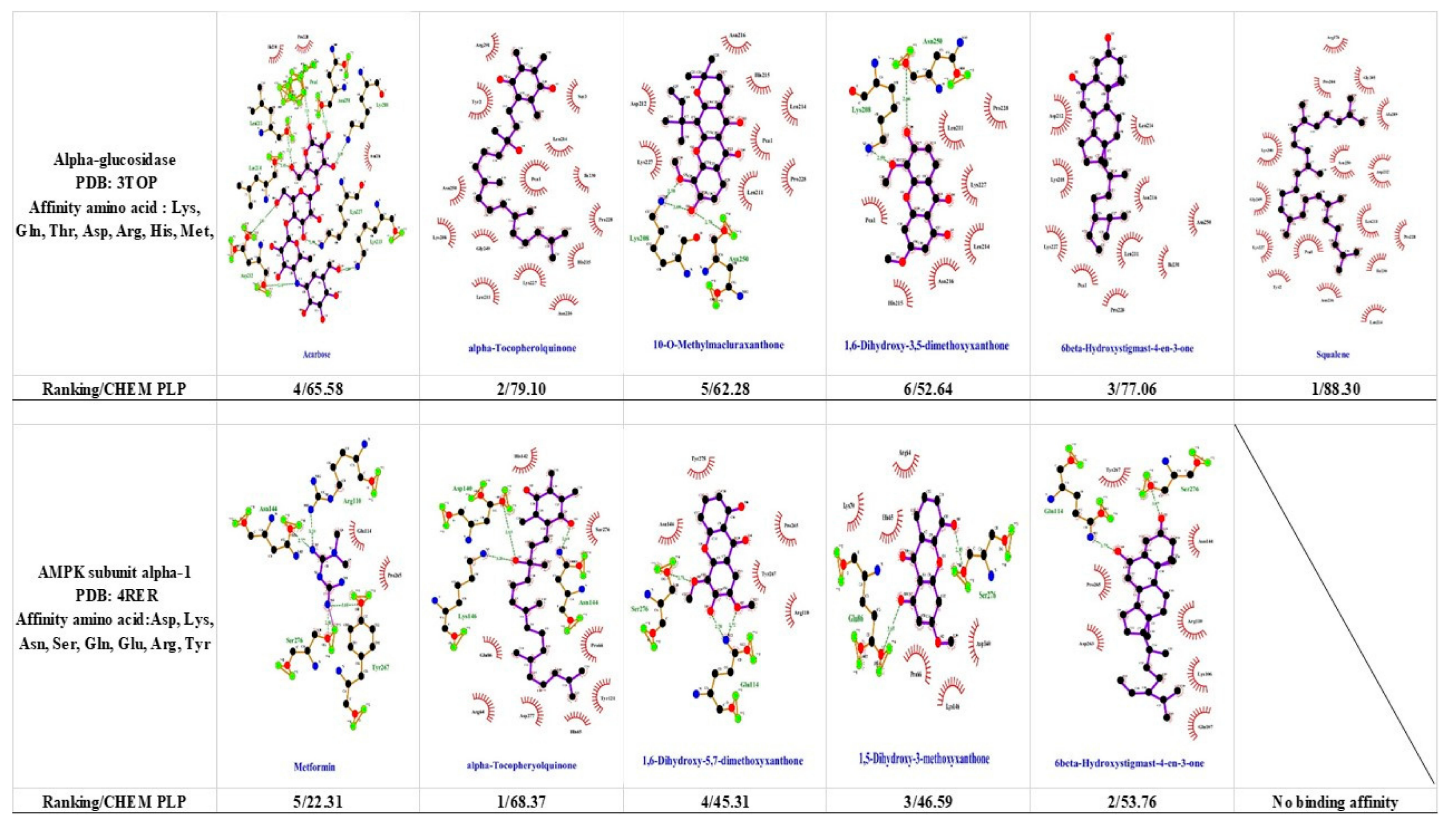

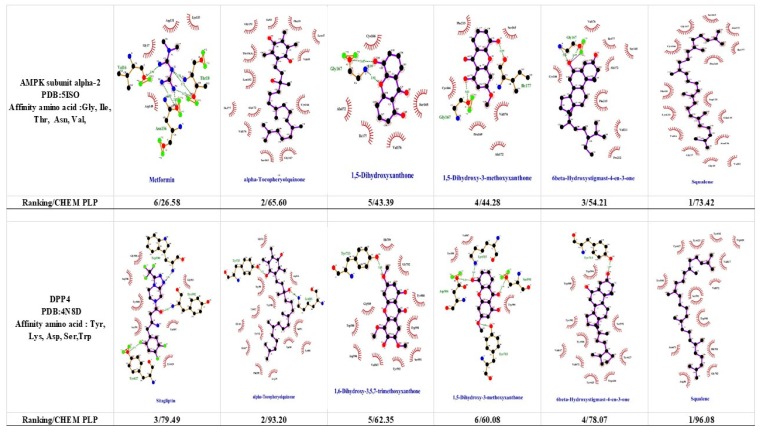

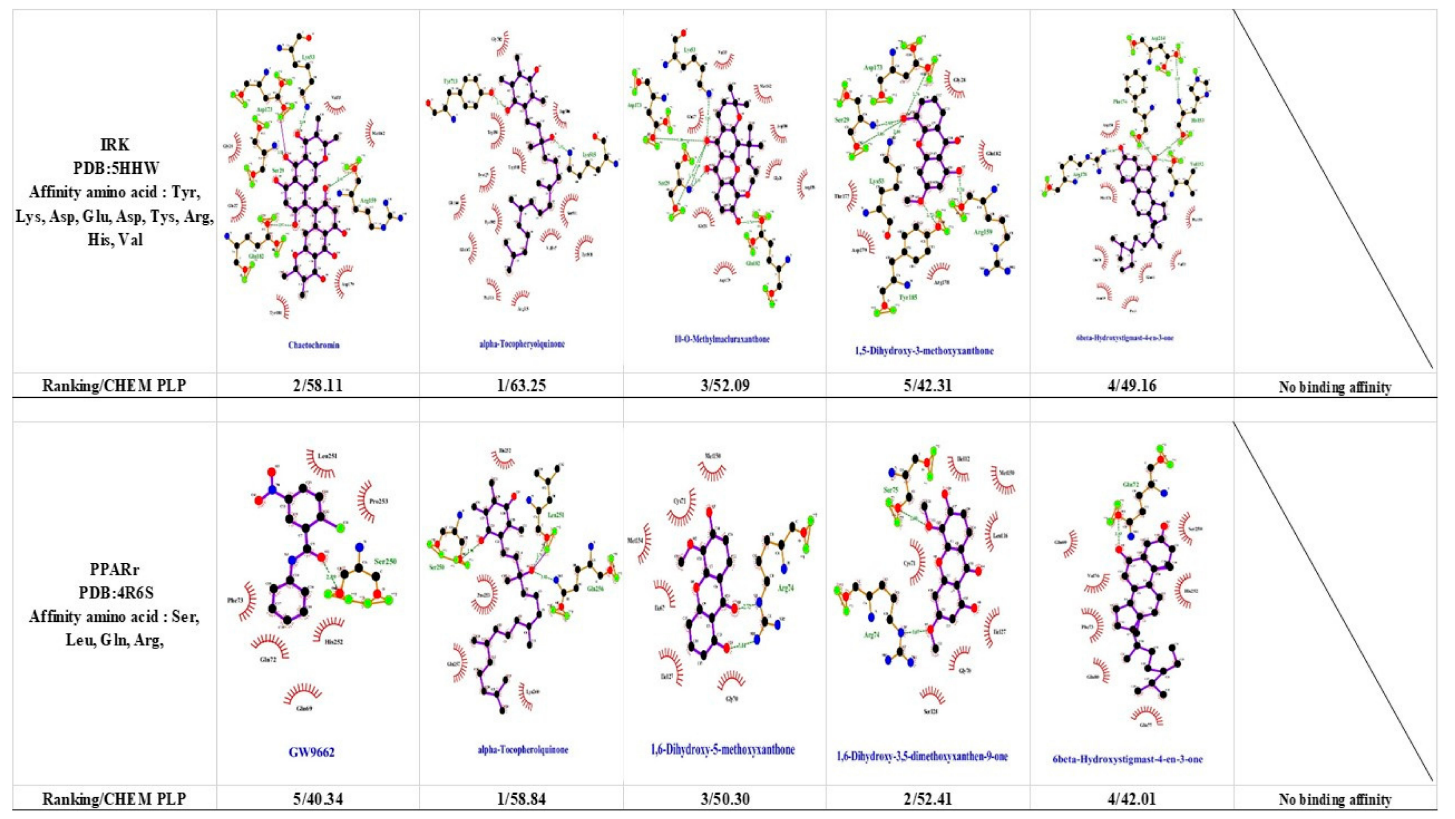
The binding affinity of benzopyrans, triterpenes, stigmastane, biflavonoid, and phenolic on α-amylase, α-glucosidase, AMPK, insulin receptor kinase, PPARγ, and DPP4. Molecular docking was performed by Gold program. Ranking/ChemPLP score presents the order of score value. The model setup was genetic algorithms (GA) run 10 times, a GA search efficiency 200%, removal of water and hydrogen, and ChemPLP scoring. ChemPLP used hydrogen bonding and multiple linear potentials to model Van der Waals and repulsive terms. α-Tocopherolquinone (a kind of benzopyrans) and squalene (a kind of triterpenes) had a higher binding affinity than the reference drug, Acarbose with α-amylase and α-glucosidase prolonging the carbohydrates hydrolyzed to reduce the absorption of glucose and regulate blood glucose levels. Interestingly, α-tocopherolquinone also had a higher binding affinity than reference drugs (Metformin, Chaetochromin, and GW9662) with AMPK1, AMPK2, PPARγ, and IRK templates, respectively; and binding signals would stimulate insulin secretion in contrast to Squalene, which only had a binding affinity with AMPK1. However, α-tocopherolquinone and Squalene still had a stronger binding affinity than Sitagliptin (reference drug) with DDP4 template that could prevent incretins from being digested by DDP4 and promote skeletal cells’ uptake of glucose from the blood.

To regulate the postprandial blood glucose level, diabetic patients took carbohydrate hydrolase inhibitors such as α-glucosidase and α-amylase to avoid hyperglycemia. α-amylase and α-glucosidase are the key enzymes to hydrolyze carbohydrates and help glucose ingestion [30]. Therefore, diabetic patients have to control their blood glucose by using clinical drugs such as *Precose^®^* (Acarbose) and *Glyset^®^* (Miglitol) [4] or other anti-diabetic natural compounds [27] to prolong hydrolysis of carbohydrates against hyperglycemia. In cumulative studies, a few crude extracts from the Garcinia species, e.g., *G. cambogia*, *G. xanthochymus*, *G. kola*, and *G. mangostana* [10,11,12,13,14], containing biflavonoids, polyphenols, and xanthones, also inhibit the enzyme activity of α-amylase and α-glucosidase. Therefore, the extracts are able to help diabetic patients to control their blood glucose levels by the inhibition of carbohydrate hydrolysis. Our docking results (Figure 1) showed that benzopyrans and triterpenes had a higher binding affinity with α-amylase and α-glucosidase than with biflavonoid and phenolic compounds. Additionally, α-tocopherolquinone (a kind of benzopyrans) and squalene (a kind of triterpenes) had a high binding affinity with α-amylase and α-glucosidase to prolong carbohydrate hydrolyzation, reduce the absorption of glucose and mediate the blood glucose level.

### 1.5. 5′-Adenosine Monophosphate-Activated Protein Kinase (AMPK)

The 5′-adenosine monophosphate-activated protein kinase (AMPK) is composed of α subunits, regulatory β subunits, and r subunits and is a sensor of cellular energy level. Cellular energy levels were changed by the ratio of AMP:ATP and ADP:ATP that influenced cellular growth and survival [31]. Previous research indicated that AMPK was activated and the rate of ATP-generating processes would increase while the rate of ATP-consuming processes decreased [32]. This mechanism has revealed that AMPK could restore energy homeostasis through an anabolic pathway to consume ATP or catabolic pathways for ATP production [33]. Hence, some clinical/reference drugs such as Metformin and Phenformin could assist peripheral tissues or skeletal muscles to uptake or utilize glucose and even increase insulin sensitivity [5,34]. To avoid adverse effects (diarrhea, nausea, ketonemia, etc.) from clinical drugs such as Metformin and Phenformin [5,35], some natural products such as curcumin [36], rutin, quercetin [37], and catechin [38] were applied to battle or ameliorate diabetes. Notably, *G. xanthochymus* in South East Asia, Africa, Australia, Thailand, and China [39] showed that it was folk medicine used for treating several diseases including diabetes. In previous studies, there were three major compounds identified: 12b-hydroxy-des-d-garcigerrin, 1,2,5,6-tretrahydroxy-4-(1,1-dimethyl-2-propenyl)-7-(3-methyl-2-butenyl) xanthone, and 1,5,6-trihydroxy-7,8-di(3-methyl-2-butenyl)-6′,6′-dimethylpyrano (2′,3′:3,4) xanthone that were isolated in the extract of *G. xanthochymus*. These compounds had a significant effect on the promotion of glucose uptake in skeletal cells when compared with Metformin [25]. Our docking results showed that α-tocopherolquinone, 6β-Hydroxystigmast-4-en-3-one, 1,6-dihydroxy-5,7-dimethoxyxanthone, 1,5-Dihydroxyxanthone, 1,5-Dihydroxy-3-methoxyxanthone, and Squalene were isolated from *G. linii* (Figure 1) and had a higher binding affinity with AMPK α1 than Metformin. Interestingly, there is a significant effect that increases glucose uptake in skeletal cells when compared with Metformin. Alternatively, *G. linii* alone or in combination with Metformin can be more prospective to alleviate side-effects or elevate applicable time (e.g., cumulative effect) by reducing Metformin dosage for clinical use.

### 1.6. Peroxisome Proliferator-Activated Receptor Gamma (PPARγ)

The peroxisome proliferator-activated receptor (PPAR) is a nuclear receptor superfamily and has three isotypes α, δ, and γ that can regulate lipid metabolism, inflammation, and insulin sensitivity as well as insulin production and secretion for treating diabetes [40,41,42]. PPARγ could mediate lipid mobilization, glucose metabolism, inflammatory response, and adipokines production and secretion [41,43]. Henceforth, cumulative studies emerged and showed PPARγ ligands that could promote triglyceride storage in fat that was implicated in insulin resistance and control adipocyte-secreted hormones [41]. In clinical treatments, Rosiglitazone is an agonist of PPARγ that could ameliorate the memory of Alzheimer patients and even increase insulin sensitivity for diabetes [44]. In traditional therapy, thiazolidinedione (TZD) was usually used to treat diabetes patients but TZD promotes triglyceride storage that causes adverse effects such as headache, muscle soreness, obesity, edema, etc. [45]. Previously, the extract of *G. cambogia* contained (−)-hydroxycitric acid (HCA), which was found to be an active ingredient used to treat obesity and obesity-related diseases, e.g., diabetes, atherosclerosis, etc. [46]. The results (Figure 1) showed that α-tocopherolquinone, 6β-Hydroxystigmast-4-en-3-one, 1,6-Dihydroxy-3,5-dimethoxyxanthen-9-one, and 1,6-Dihydroxy-5-methoxyxanthone stimulated insulin sensitivity, and in virtual screening via the binding affinity of GW9662 (reference drug), which is lower than those of compounds isolated from *G. linii*.

### 1.7. Dipeptidyl-Peptidase 4 (DPP-4) and Glucagon-Like Peptide 1 (GLP-1)

The dipeptidyl peptidase-4 (DPP-4) could hydrolyze glucagon-like peptide 1 (GLP-1) or gastric inhibitory polypeptide (GIP) and lead to negative effects on the concentration of incretins (GLP-1 and GIP), insulin secretion, and glucose tolerance due to DPP4 gene expression [47]. Consequently, some diabetes patients may take a DPP4 inhibitor such as Sitagliptin to increase insulin secretion for diabetes therapy and ameliorate the therapeutic effect of GLP-1 [48,49]. The GLP-1 was treated with DPP4 inhibitors against diabetes from 2005 to 2007 and still had adverse effects such as rhinopharyngitis and upper respiratory tract infections [50]. Therefore, some cumulative studies indicated that natural compounds, e.g., rutin, curcumin, antroquinonol, quercetin, and 16-hydroxy-cleroda-3, 13-dien-15, 16-olide (HCD), could inhibit DPP4 activity, such as the inhibitory efficacy of curcumin and quercetin, better than Sitagliptin [36,37,51]. A previous report showed that the extract of the *G. cambogia* fruit, which contains hydroxycitric acid (HCA), could decrease the serum insulin levels and prolong intestinal tracts to absorb glucose as well as to potentially change incretins (GLP-1, GIP) secretions [10,52]. Taken altogether, the extract of *G. cambogia* could regulate blood glucose levels, treat metabolic syndromes, and lead to weight loss. To increase insulin sensitivity, our docking results showed that benzopyrans, triterpenes, stigmastane, and biflavonoids were found to act as insulin receptor agonists and promoted glucose uptake in skeletal cells from blood. Hereafter, incretins are degraded by DPP4 and lead to pancreatic β cells to decrease secretions of insulin (Figure 1). Of note, the reference drug, Sitagliptin, plays a major role in inhibiting the activation of DPP4. Obviously, our data indicated that α-tocopherolquinone and squalene had stronger binding affinity with DPP4 as an inhibitor than with Sitagliptin to prevent incretin (GLP-1) degraded by DPP4.

### 1.8. Insulin Receptor Kinase (IRK)

α-subunits of insulin receptors receive signal insulin, which triggers tyrosine kinase of β-subunits (Insulin receptor kinase, IRK) to form intracellular auto-phosphorylation at Tyr1158, Tyr1162, and Tyr1163 [53]. Once the insulin receptors are activated, they promote PI3K to phosphorylate PIP2; and, further, PIP3 leads the PDK1/2 activation. When AKT was phosphorylated by receiving the signal, the downstream AS160 would prompt glucose transporter 4 (GLUT4) translocation and uptake glucose into the cells [54]. Previously, some natural compounds have been demonstrated such as (+)-antroquinonol isolated from *Antrodia cinnamomea* [55], rutin (a kind of flavonoid) isolated from *Toona sinensis* Roem [53], and the phenolics isolated from coffee silverskins and husks [56] that result in lowered glucose levels. All of these compounds could enhance the activation of IRK to promote the skeletal tissues to absorb glucose and, consequently, ameliorate insulin resistance by reducing blood glucose levels in the diabetic patients. Therefore, in this study, we collated research from the literature by the application of the Garcinia species for various anti-diabetes treatments. Previous literature revealed that *G. xanthochymus, G. kola, G. mangostana, G. pedunculata*, and *G. prainiana* contained natural compounds, e.g., biflavonoids, xanthone, HCA, and depsidone, which could augment IRK activity and regulate the blood glucose levels for diabetic patients [10,15,16,25,57]. Accordingly, our docking data revealed that only α-tocopherolquinone had a higher binding affinity with IRK than a reference drug (Chaetochromin), suggesting that α-tocopherolquinone acts as an anti-hyperglycemic compound to heighten IRK activity (Figure 1).

## 2. Conclusions and Future Remarks

In ancient societies, the Garcinia species were used as a daily supply, e.g., building material, food additives, fruit juice, jam, and dye. However, natural compounds that are isolated from the bark, seeds, fruits, peels, leaves, and stems of some Garcinia species such as *G. kola*, *G. pedunculata, G. prainiana, G. mangostana, G. xanthochymus*, and *G. cambogia* have been reported to have a variety of medicinal values. These compounds are applied to treat adipogenesis, inflammation, obesity, cancer, cardiovascular diseases, and diabetes. Predominantly, the isolated natural compounds of *G. linii* in this study are employed to do molecule docking with α-amylase, α-glucosidase, AMPK, IRK, PPARγ, and DPP4, respectively. Of note, our docking data revealed that the ChemPLP scores for Benzopyrans, Flavonols, Polyphenol, Stigmastane, and Triterpenes isolated from *G. linii* had a higher AMPK affinity when compared with Metformin; and, alternatively, *Garcinia linii* alone or in combination with Metformin can have a greater potential to alleviate side-effects or elevate applicable time (e.g., cumulative effect) by reducing Metformin dosage. These results demonstrated that benzopyrans and triterpenes had a stronger binding affinity with anti-diabetic target molecules as a template than reference drugs, e.g., Acarbose with α-Amylase and α-Glucosidase, Metformin with AMPK, Sitagliptin with DPP4, Chaetochromin with IRK, and GW9662 with PPARγ. According to this evidence, benzopyrans and triterpenes are suggested to be the active components in *G. linii* for mediating blood glucose. To further validate the potency of these active components, compounds purified and subsequently the enzyme activity test, an *in vitro* cellular function assay and an *in vivo* animal efficacy experiment need to be conducted to investigate their potential role in anti-diabetes and anti-hyperglycemia in the future.

## Figures and Tables

**Table 1:** Summary of the Garcinia species on specific targets of anti-diabetes with basic findings.

Species.	Molecular Targets	Basic Findings
*G. cambogia*	α-Glucosidase, PPARγ, DPP4	Small intestinal exposure to HCA resulted in a modest reduction in glycemia of healthy individuals [20]. Mixture (GE containing HCA as an active ingredient, PE, anti-adipogenic activity) reduced the expression of adipogenesis-related factors C/EBP-α, PPARγ, and FAS [46]. Insulin resistance did not develop in HCA-SX-supplemented rats via lowered fasting plasma insulin and glucose [58,59].
*G. xanthochymus*	α-Amylase, α-Glucosidase, AMPK, IRK	Activated PI3K/PKB/Akt signaling pathway and AMPK signaling pathway, resulting in the translocation of GLUT4 in L6 myotubes without affecting the expression of GLUT4 [18]. Identification of α-amylase inhibitor from *G. xanthochymus* [21].
*G. kola*	α-Amylase, IRK	KV offered significant anti-diabetic relief via reduction of FBG, α-amylase and HbA1c [22].
*G. mangostana*	α-Amylase, IRK	MVR from *G. mangostana* fruit pericarp had an α-amylase inhibitor and enhanced insulin sensitivity [23,24] GME significantly reduced the blood glucose level in normoglycemic rats and STZ-induced diabetic rats [57].
*G. pedunculata*	IRK	Elevated insulin levels of rats [25].
*G. prainiana*	IRK	Increased insulin sensitivity of 3T3-L1 adipocytes [26].

*G. cambogia* extract (GE); (−)-hydroxycitric acid (HCA); *Pear pomace* extract (PE), CCAAT-enhancer binding protein alpha (C/EBP-α); Peroxisome proliferator-activated receptor gamma (PPARγ); fatty acid synthase (FAS); insulin receptor kinase (IRK); phosphatidylinositol-3 kinase (PI3K)/the serine/threonine kinase protein kinase B (PKB/Akt); AMP-activated protein kinase (AMPK); fasting blood glucose (FBG); kolaviron (KV); mangosteen vinegar rind (MVR); Super CitriMax hydroxycitric acid (HCA-SX), a novel calcium/potassium salt; *G. mangostana* pericarp ethanolic extract (GME); glycated hemoglobin (HbA1c); Streptozotocin (STZ).
